# JAK Inhibition for the Treatment of Myelofibrosis: Limitations and Future Perspectives

**DOI:** 10.1097/HS9.0000000000000424

**Published:** 2020-07-21

**Authors:** Prithviraj Bose, Srdan Verstovsek

**Affiliations:** Department of Leukemia, University of Texas MD Anderson Cancer Center, Houston, Texas, USA.

## Abstract

The 2011 approval of ruxolitinib ushered in the Janus kinase (JAK) inhibitor era in the treatment of myelofibrosis (MF), and 2019 saw the US approval of fedratinib. The first therapeutic agents approved by regulatory authorities for MF, these drugs attenuate the overactive JAK-signal transducer and activator of transcription (STAT) signaling universally present in these patients, translating into major clinical benefits in terms of spleen shrinkage and symptom improvement. These, in turn, confer a survival advantage on patients with advanced disease, demonstrated in the case of ruxolitinib, for which long-term follow-up data are available. However, JAK inhibitors do not improve cytopenias in most patients, have relatively modest effects on bone marrow fibrosis and driver mutation allele burden, and clinical resistance eventually develops. Furthermore, they do not modify the risk of transformation to blast phase; indeed, their mechanism of action may be more anti-inflammatory than truly disease-modifying. This has spurred interest in rational combinations of JAK inhibitors with other agents that may improve cytopenias and drugs that could potentially modify the natural history of MF. Newer JAK inhibitors that are distinguished from ruxolitinib and fedratinib by their ability to improve anemia (eg, momelotinib) or safety and efficacy in severely thrombocytopenic patients (eg, pacritinib) are in phase 3 clinical trials. There is also interest in developing inhibitors that are highly selective for mutant JAK2, as well as “type II” JAK2 inhibitors. Overall, although current JAK inhibitors have limitations, they will likely continue to form the backbone of MF therapy for the foreseeable future.

## Introduction

The discovery in 2005 of the activating V617F mutation in Janus kinase 2 (JAK2) in the majority of patients with classic Philadelphia chromosome negative myeloproliferative neoplasms (MPNs)^1^,^2^,^3^,^4^ led to the development of small-molecule inhibitors of the JAK family of tyrosine kinases, culminating in the regulatory approval of ruxolitinib in 2011 for the treatment of myelofibrosis (MF).^5^ Over the years, both the benefits and limitations of JAK inhibitor therapy have become apparent. Both ruxolitinib and fedratinib (approved in the US in 2019) provide robust clinical benefits to patients in terms of spleen volume reduction (SVR) and symptomatic improvement. However, both cause substantial anemia and thrombocytopenia, especially early on in therapy, and neither is recommended for use in patients with baseline platelets <50 × 10^9^/L. Additional concerns with fedratinib include gastrointestinal toxicity and a potential to cause Wernicke encephalopathy, albeit rarely. The experience with ruxolitinib is much more extensive than that with fedratinib: 5-year follow-up of the pivotal COMFORT trials reveals an overall survival (OS) advantage for patients randomized to ruxolitinib, despite crossover, and a median duration of spleen response of approximately 3 years; 15.8% of ruxolitinib-randomized patients in COMFORT-2 had improved bone marrow fibrosis after a median duration of treatment of 2.2 years and the allele burden of mutant *JAK2* had declined by >20% in 31% of patients at week 192.^6^,^7^,^8^ Ruxolitinib, while generally very well-tolerated, is immunosuppressive and may precipitate opportunistic infections.^9^,^10^ The risk of non-melanoma skin cancer with ruxolitinib is also well-established.^11^ The reported increased risk of aggressive B-cell non-Hodgkin lymphoma (NHL) with JAK inhibitor use in patients with MF^12^ appears largely unfounded, both based on our experience^13^ and that of Italian investigators.^14^ Patients with MPN are at increased risk for second malignancies (including NHL)^15^,^16^,^17^; in a recent, large, nested case-control study, mortality from the second cancer (SC) was associated with age >70 years, type of SC, relapse of the SC, MPN evolution, anemia at SC diagnosis, and exposure to hydroxyurea and to ruxolitinib.^18^

## Ruxolitinib

The JAK1/2 inhibitor ruxolitinib, licensed for the treatment of MF in 2011, was the first drug specifically approved for this condition and has since become the cornerstone of MF therapy. In the pivotal, phase 3 COMFORT-1 and -2 trials conducted in patients with intermediate-2 or high risk MF and baseline platelets ≥100 x 10^9^/L, ruxolitinib led to ≥35% SVR in 42% and 32% of patients at week 24, respectively; additionally, in COMFORT-1, 46% of the patients in the ruxolitinib arm experienced a ≥50% improvement in total symptom score (TSS) at 24 weeks.^19^,^20^ While neither trial was powered to demonstrate a difference in OS, an exploratory analysis of 5-year data pooled from both trials showed a 30% reduction in the risk of death among patients randomized to ruxolitinib (median OS, 5.3 vs 3.8 years) compared with patients in the control (placebo in COMFORT-1 and best available therapy (BAT) in COMFORT-2) group.^6^ Using the rank preserving structural failure time (RPSFT) technique to correct for the universal crossover that occurred in both trials, the difference was even more pronounced (5.3 vs 2.3 years). This survival benefit of ruxolitinib seen in the COMFORT trials has been a contentious subject as the trials were not designed with OS as a primary endpoint, patients with baseline platelets <100 × 10^9^/L (who have a worse outcome)^21^ were excluded, and patients with post-polycythemia vera/essential thrombocythemia MF (who have a better prognosis)^22^ might have been over-represented.^23^,^24^,^25^ While subgroup analyses of the COMFORT trials failed to reveal any predictive factors for benefit from ruxolitinib, that is, benefit was seen across subgroups,^26^ other “real-world” analyses have identified intermediate-2/high risk disease, large splenomegaly, transfusion dependence (TD), platelets <200 × 10^9^/L and a >2 year interval between MF diagnosis and ruxolitinib initiation as negatively correlating with spleen response to ruxolitinib.^27^ Multiple studies have shown that spleen responses to ruxolitinib are dose-dependent and correlate with survival,^28^,^29^,^30^ arguing for the importance of dose intensity early in therapy, although a more conservative dosing strategy in anemic patients (10 mg twice daily during the first 12 weeks, followed by escalation) may also be reasonable.^31^ Anemia induced by ruxolitinib does not carry the adverse prognosis of disease-associated anemia^32^; indeed, ruxolitinib has been shown to overcome the deleterious prognostic impact of the latter.^33^ The durability of spleen response to ruxolitinib has also been shown to influence patient outcomes.^34^ While the ruxolitinib label suggests a starting dose of 5 mg twice daily in patients with baseline platelets 50–99 × 10^9^/L, studies support using the more effective starting dose of 10 mg twice daily in this population.^35^,^36^ Finally, although only intermediate-2 and high risk patients were included in the COMFORT trials, a large body of data supports the use of ruxolitinib in patients with intermediate-1 risk disease, in whom it may be more effective and less toxic.^37^,^38^,^39^,^40^ While technically not approved in the US for use in low risk patients, consensus guidelines from the National Comprehensive Cancer Network support the use of ruxolitinib under certain circumstances, such as in symptomatic, low risk patients.^41^ However, guidelines from the European LeukemiaNet and the Italian Society of Hematology do not recommend the use of ruxolitinib with a goal of improving survival in the absence of significant splenomegaly or symptoms.^42^ Lipid levels should be checked at ruxolitinib initiation and periodically during treatment, and treatment instituted if appropriate. In our practice, we routinely vaccinate patients receiving ruxolitinib with the inactivated shingles vaccine, and institute indefinite prophylaxis with acyclovir/valacyclovir following an episode of shingles. We also check serologies for evidence of hepatitis B and C infection before beginning ruxolitinib, and obtain infectious disease consultation for patients who have active infection or evidence of prior/latent hepatitis B infection (negative HBsAg but positive anti-HBc antibody). Although prospective data are scarce, our preference is to perform allogeneic hematopoietic cell transplantation (allo-HCT) in patients in whom it is appropriate at the time of best response to ruxolitinib. In line with consensus recommendations, we continue ruxolitinib until the day prior to commencement of conditioning, and taper the dose over 5 to 7 days.^43^ Data in small numbers of patients suggest that it may be possible to continue ruxolitinib through allo-HCT without adversely impacting engraftment, and peri-transplant use of ruxolitinib may prevent acute graft vs host disease (aGVHD) in patients with MF undergoing allo-HCT.^44^,^45^ Ruxolitinib's recent approval for steroid-refractory aGVHD is likely to increase use of this agent in the post-transplant setting.^46^ Ruxolitinib has also shown promise in the treatment of steroid-refractory chronic graft vs host disease (cGVHD).^47^,^48^

While the clinical benefits of ruxolitinib are undeniable, the drug clearly has some limitations. Ruxolitinib appears most efficacious in patients with a *JAK2* V617F allele burden >50%,^49^ and patients with genetically complex disease (as evidenced by the presence of non-driver mutations in *ASXL1*, *DNMT3A* or *EZH2* and, in particular, those with ≥3 non-driver mutations) have substantially lower odds of spleen response and inferior OS.^50^ As alluded to above, the benefits of ruxolitinib in terms of bone marrow fibrosis reduction and evidence of its anti-clonal activity are modest; of 236 *JAK2* V617F+ patients in COMFORT-1, 20 and 6 achieved partial and complete molecular responses (CMR), with median times to response of 22.2 and 27.5 months, respectively.^51^ Clinical resistance to ruxolitinib may be due to the “persistence” phenomenon, whereby JAK2 is transactivated via heterodimerization with another member of the JAK family despite the presence of the inhibitor.^52^ Patients who discontinue ruxolitinib have a dismal outcome with median OS 13 to 14 months, with those with clonal evolution and/or dropping platelet counts on ruxolitinib doing particularly poorly.^53^,^54^,^55^ Anemia is the most frequent reason for discontinuation of ruxolitinib in clinical practice^54^ and represents a significant practical challenge. Similarly, ruxolitinib is difficult to use in patients with severe thrombocytopenia (platelets <50 × 10^9^/L), a major hallmark of the so-called “myelodepletive phenotype” in MF.^56^

## Fedratinib

Development of the JAK2 inhibitor fedratinib was halted despite positive results in the phase 3, placebo-controlled JAKARTA trial in patients with intermediate-2/high risk MF and baseline platelets ≥50 × 10^9^/L after concerns over Wernicke encephalopathy (WE) led the US Food and Drug Administration (FDA) to place a full clinical hold on all trials of fedratinib.^57^ The efficacy and hematologic toxicity profile of fedratinib is similar to that of ruxolitinib: in JAKARTA, 36% of patients randomized to receive 400 mg daily of fedratinib (the currently approved dose) achieved ≥35% SVR (confirmed 4 weeks later) and ≥50% reduction in TSS at week 24. Because of fedratinib's inhibitory effect on fms-like tyrosine kinase 3 (FLT3), gastrointestinal toxicity (nausea, vomiting, diarrhea) is significant. The 8 putative cases of WE (occurring in 670 patients treated across the fedratinib development program) were later re-analyzed, and only 1 was deemed to be a confirmed case, while 2 others likely had WE, although neurodeficits recovered in these 2 patients despite continued fedratinib treatment.^58^ The diagnosis was inconclusive in 2 other patients, while 3 other patients did not appear to have WE. These findings, along with the JAKARTA data, served as the basis of the FDA approval of fedratinib for the treatment of MF in 2019. Nevertheless, WE is the subject of a black box warning in the US prescribing information for fedratinib, and thiamine levels are recommended to be checked (and any deficiency corrected) prior to fedratinib initiation and periodically during treatment. Fedratinib should be stopped immediately and parenteral thiamine supplementation begun upon suspicion of encephalopathy. JAKARTA-2 was a single-arm, open-label study of fedratinib, 400 mg daily, in 97 ruxolitinib-exposed (minimum 14 days, median 10.7 months) patients with MF; this trial was terminated prematurely because of the clinical hold, resulting in missing week 24 data in a number of patients.^59^ By intention-to-treat (ITT) analysis, the rate of ≥35% SVR at 24 weeks was 31%, and the rate of ≥50% TSS reduction 27%, rates that remained virtually unchanged upon re-analysis of the data using “stringent” criteria for ruxolitinib failure (see Table 1) that were fulfilled by 79 of the 97 patients (24-week rates of ≥35% SVR and ≥50% TSS reduction in the stringent criteria cohort 30% and 27%, respectively).^60^ Although approved in the US, fedratinib is now being studied in the post-ruxolitinib setting as defined by these criteria in the FREEDOM trials in the US and Europe. These trials will be critical to gain long-term experience with fedratinib in MF patients, such data being unavailable from the JAKARTA trials. Of note, the efficacy of fedratinib appears to not differ significantly based on the baseline platelet count (50–99 × 10^9^/L vs ≥100 × 10^9^/L) and the recommended dose is the same (400 mg daily) in both platelet count subgroups, unlike the case with ruxolitinib.^61^

**Table 1:**
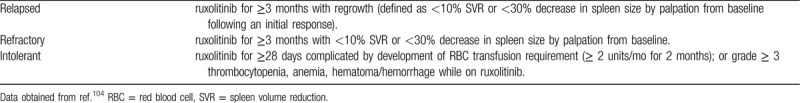
Criteria for Ruxolitinib Failure Used in the Re-analysis of JAKARTA-2,^60^ PAC203^71^ and FREEDOM Trials.

## Momelotinib

Momelotinib is a JAK1/2 inhibitor that appears to improve anemia.^62^,^63^ The mechanism behind this unexpected benefit of a JAK2 inhibitor was poorly understood until momelotinib was shown to down-regulate hepatic hepcidin production via antagonism of the type 1 activin receptor (ACVR1/ALK2) and ameliorate anemia in a rodent model of anemia of chronic disease.^64^ In the phase 3, head-to-head SIMPLIFY-1 trial conducted in the JAK inhibitor naïve setting, momelotinib was non-inferior to ruxolitinib for ≥35% SVR at 24 weeks (26.5% vs 29%) but not for ≥50% TSS reduction (28% vs 42%).^65^ The phase 3 SIMPLIFY-2 trial, which compared momelotinib to BAT in ruxolitnib-exposed patients, did not meet its primary endpoint of ≥35% SVR at week 24; notably, in this trial, 88% of patients in the BAT arm received ruxolitinib.^66^ Nevertheless, symptom improvement with momelotinib was noteworthy (26% rate of ≥50% TSS reduction at 24 weeks, compared to 6% with BAT), although statistical significance could not be claimed because of the primary endpoint not having been met. In both trials, the anemia-related endpoints all favored momelotinib, but again, the hierarchical design precluded formal statistical testing. Momelotinib will now be compared to danazol (2:1) in 180 ruxolitinib-pretreated patients with MF in the phase 3 MOMENTUM trial, which has a primary endpoint of ≥50% TSS reduction at week 24, with achievement of transfusion independence (TI) and ≥35% SVR at week 24 being key secondary endpoints.

## Pacritinib

The JAK2/FLT3 inhibitor pacritinib is relatively non-myelosuppressive and trials have not specified a minimum platelet count threshold for eligibility.^67^,^68^ Pacritinib beat BAT (excluding JAK inhibitors) in JAK inhibitor-naïve patients in the phase 3 PERSIST-1 trial for the primary endpoint of ≥35% SVR at 24 weeks (19% vs 5%), while for ≥50% TSS reduction, the difference in the ITT population was not statistically significant at 24 weeks, although it was at 48 weeks.^69^ Importantly, the superiority of pacritinib over BAT was maintained in the subgroups of patients with baseline thrombocytopenia (platelets <100 × 10^9^/L and <50 × 10^9^/L). The phase 3 PERSIST-2 trial compared 2 doses of pacritinib, 400 mg once daily and 200 mg twice daily, against BAT, which could be a JAK inhibitor, and was in 45% of the patients.^70^ Approximately 45% of patients had received prior JAK inhibitor therapy, and eligibility was restricted to thrombocytopenic patients (baseline platelets <100 × 10^9^/L). Pacritinib (arms combined) was superior to BAT for ≥35% SVR at 24 weeks (18% vs 3%) but not for the co-primary endpoint of ≥50% TSS reduction (25% vs 14%), although it was at the 200 mg twice daily dose, for both measures. Like JAKARTA-2, analysis of this trial was also impacted by the placement of a full clinical hold by the FDA on pacritinib trials owing to concerns over excess mortality from bleeding and cardiac arrhythmias. This hold was subsequently lifted and a dose-finding study (PAC203) conducted in 161 patients meeting the stringent criteria for “ruxolitinib failure” set forth in Table 1, and incorporating a number of risk mitigation strategies.^71^ The 200 mg twice daily dose emerged as the winner from this study, albeit with very modest rates of ≥35% SVR (9.3%) and ≥50% TSS reduction (7.4%) at 24 weeks. Of interest, 17% of patients with baseline platelets <50 × 10^9^/L receiving 200 mg twice daily achieved ≥35% SVR at 24 weeks. This trial has now been amended to a phase 3 trial, PACIFICA, in which pacritinib will be compared against physician's choice treatment (low dose ruxolitinib, steroids, hydroxyurea, danazol) in MF patients with baseline platelets <50 × 10^9^/L who are JAK inhibitor-naïve or have received up to 90 days of prior JAK inhibitor therapy.

## Ruxolitinib-based combination approaches

Given that ruxolitinib is the mainstay of therapy for MF and confers a survival benefit in patients with intermediate/high risk disease, there has been much interest developing ruxolitinib-based rational combinations to counteract cytopenias and to obtain deeper responses (most responses to ruxolitinib monotherapy are in the “clinical improvement” (CI) category – complete and partial responses (PRs) are rare). With regards to anemia, the activin receptor ligand trap luspatercept, recently approved for the treatment of anemia in patients with lower risk myelodysplastic syndromes and ring sideroblasts,^72^ has shown promising activity, particularly in transfusion-dependent patients on a stable dose of ruxolitinib,^73^ and a phase 3 trial is planned. Low dose thalidomide (50 mg daily) in conjunction with ruxolitinib appears to be a promising strategy to counteract thrombocytopenia^74^ and could allow ruxolitinib to be dosed more safely in patients with platelets <50 × 10^9^/L, but needs to be studied in more patients, including specifically this subgroup. Lenalidomide is difficult to administer along with ruxolitinib because of excessive myelosuppression,^75^ while pomalidomide continues to be studied,^76^ primarily as a treatment for anemia, despite earlier negative results in the phase 3, placebo-controlled setting as a single agent.^77^ Taking a cue from momelotinib, an ACVR1/ALK2 inhibitor, INCB00928, will be studied both alone and in combination with ruxolitinib.

A number of potentially disease-modifying ruxolitinib-based combinations, some based on preclinical evidence of synergism, have been studied in clinical trials. Combinations with hedgehog pathway (Smoothened) inhibitors,^78^ histone deacetylase inhibitors^79^,^80^ and pan-phosphatidylinositol-3-kinase (pan-PI3K) inhibitors,^81^ although promising in the laboratory,^82^,^83^,^84^ proved disappointing in the clinic, while intriguing results were reported in an investigator-initiated trial of a more empiric combination, that of ruxolitinib with azacitidine.^85^ Although the lack of a ruxolitinib-alone control group and spleen response assessment by palpation were major confounders, responses to the combination appeared superior to those expected with ruxolitinib alone. Interestingly, improvement in bone marrow fibrosis was reported in 60% of patients at 12 months. Laboratory-based rational combinations^82^,^86^,^87^ currently in clinical trials with early results in the public domain include those of ruxolitinib with the PI3K delta isoform inhibitors parsaclisib^88^ and umbralisib,^89^ the bromodomain and extra-terminal (BET) protein inhibitor CPI-0610^90^ and the Bcl-2/-xL antagonist, navitoclax.^91^ These agents have been studied in “add-on” fashion in patients with “sub-optimal” responses (variously defined) to ruxolitinib monotherapy (Table 2). Notwithstanding important differences in eligibility criteria and baseline patient characteristics, CPI-0610 and navitoclax appear the most promising partners for ruxolitinib at present. Interestingly, response rates (spleen, symptoms, anemia (TI)) to the ruxolitinib/CPI-0610 combination were much higher in transfusion-dependent patients, a poorly understood phenomenon akin to that observed in the luspatercept trial^73^ mentioned above. Both the CPI-0610 and navitoclax combinations with ruxolitinib are also being studied in the JAK inhibitor-naïve setting; early results with the former are promising (10 of 15 (66.7%) evaluable patients achieved ≥35% SVR and 11 of 14 (78.6%) evaluable patients achieved ≥50% TSS reduction at 24 weeks)^92^ and a randomized phase 3 trial of the combination compared to ruxolitinib alone in JAK inhibitor-naïve patients has been announced. However, the current dosing of ruxolitinib in the combination (one dose level lower than would be indicated by the platelet count to minimize myelosuppression) could make the design and interpretation of a randomized phase 3 trial problematic.

**Table 2:**
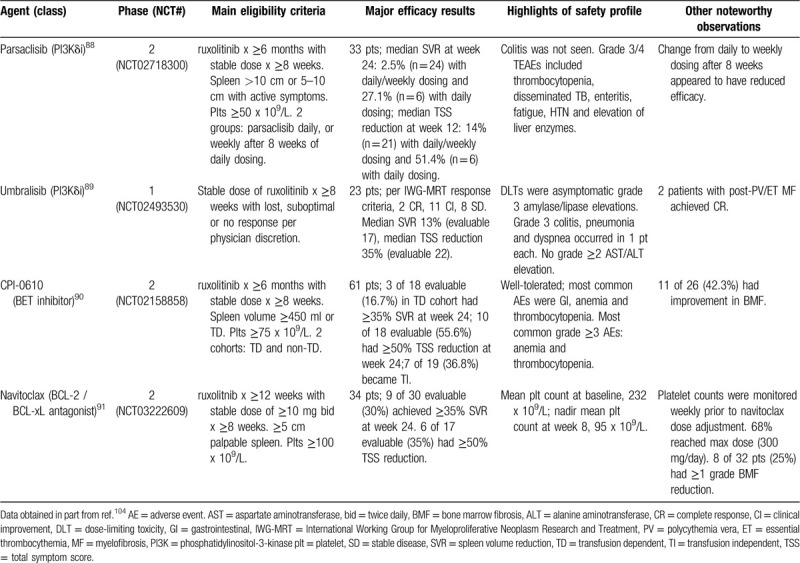
“Add-on” Approaches to Ruxolitinib Being Studied in Ongoing Clinical Trials With Results Available in the Public Domain.

Yet other novel agents are being explored in combination with ruxolitinib in clinical trials, most of them employing an “add on” strategy; examples include the heat shock protein 90 inhibitor PU-H71 (NCT03935555), the protein neddylation inhibitor pevonedistat (NCT03386214), the PIM kinase inhibitor (NCT02587598), and the JAK1 inhibitor itacitinib (NCT03144687). Others are expected to enter clinical trials soon, eg, poly (ADP-ribose) polymerase inhibitors, mitogen activated protein kinase inhibitors and inhibitors of cyclin-dependent kinases 4/6 (personal communications, Steffen Koschmieder and Raajit Rampal). An impressive body of preclinical work supports most of these approaches.^93^,^94^,^95^,^96^,^97^,^98^

## The quest for “type 2” and mutant-specific JAK2 inhibitors

As noted above, one mechanism of clinical resistance to type 1 JAK2 inhibitors, which bind to and stabilize the kinase in its active conformation (all JAK2 inhibitors currently in the clinic) may be the phenomenon of “persistence”, which can be reversed in the laboratory by withdrawal of the drug^52^; as a clinical correlate, withdrawal of ruxolitinib followed by re-challenge has been reported to restore sensitivity to the drug.^99^ CHZ868 is a type 2 JAK2 inhibitor that binds to the inactive conformation of the kinase and can reverse type 1 JAK2 inhibitor persistence.^100^ However, this drug is not a clinical candidate.

There is also much interest in the development of mutant-specific inhibitors of JAK2 that should, in theory, spare wild type JAK2 and avoid on-target toxicities such as anemia and thrombocytopenia, among others. NS-018, a JAK2 inhibitor that has been tested in the clinic, is weakly selective for *JAK2* V617F over wild type *JAK2* ^101^; however, the clinical efficacy of this agent is modest and its toxicity profile not significantly different in terms of myelosuppression^102^; at this time, the developmental path forward for NS-018 remains uncertain. Recent structure-guided mutagenesis studies have provided key insights that may inform the development of highly selective inhibitors of *JAK2* V617F.^103^

## Conclusions

JAK inhibitors have emerged as the centerpiece of pharmacologic therapy for patients with MF, providing unprecedented benefits in terms of spleen shrinkage, symptom improvement and quality of life that can enhance longevity in patients with advanced disease. There is also considerable evidence that ruxolitinib is more efficacious when initiated earlier in the disease process.^27^,^37^,^38^,^51^ As such, continued efforts to develop JAK inhibitors with improved characteristics over the two currently registered makes sense, as does the development of novel, ruxolitinib- and fedratinib-based combinations. Although many agents with distinct mechanisms of action are being explored as monotherapy after “failure” of ruxolitinib (reviewed in Ref. ^104^), it appears that several of these will also be best positioned as partners for JAK inhibitors.
